# Mevalonate Cascade Regulation of Airway Mesenchymal Cell Autophagy and Apoptosis: A Dual Role for p53

**DOI:** 10.1371/journal.pone.0016523

**Published:** 2011-01-31

**Authors:** Saeid Ghavami, Mark M. Mutawe, Pawan Sharma, Behzad Yeganeh, Karol D. McNeill, Thomas Klonisch, Helmut Unruh, Hessam H. Kashani, Dedmer Schaafsma, Marek Los, Andrew J. Halayko

**Affiliations:** 1 Department of Physiology, University of Manitoba, Winnipeg, Manitoba, Canada; 2 National Training Program in Allergy and Asthma, University of Manitoba, Winnipeg, Manitoba, Canada; 3 Department of Human Anatomy and Cell Science, University of Manitoba, Winnipeg, Manitoba, Canada; 4 Department of Internal Medicine, University of Manitoba, Winnipeg, Manitoba, Canada; 5 Biology of Breathing Group, Manitoba Institute of Child Health, Winnipeg, Manitoba, Canada; 6 Department of Clinical & Experimental Medicine, Integrative Regenerative Medical Center (IGEN), Linköping University, Linkoping, Sweden; Wayne State University School of Medicine, United States of America

## Abstract

Statins inhibit the proximal steps of cholesterol biosynthesis, and are linked to health benefits in various conditions, including cancer and lung disease. We have previously investigated apoptotic pathways triggered by statins in airway mesenchymal cells, and identified reduced prenylation of small GTPases as a primary effector mechanism leading to p53-mediated cell death. Here, we extend our studies of statin-induced cell death by assessing endpoints of both apoptosis and autophagy, and investigating their interplay and coincident regulation. Using primary cultured human airway smooth muscle (HASM) and human airway fibroblasts (HAF), autophagy, and autophagosome formation and flux were assessed by transmission electron microscopy, cytochemistry (lysosome number and co-localization with LC3) and immunoblotting (LC3 lipidation and Atg12-5 complex formation). Chemical inhibition of autophagy increased simvastatin-induced caspase activation and cell death. Similarly, Atg5 silencing with shRNA, thus preventing Atg5-12 complex formation, increased pro-apoptotic effects of simvastatin. Simvastatin concomitantly increased p53-dependent expression of p53 up-regulated modulator of apoptosis (PUMA), NOXA, and damage-regulated autophagy modulator (DRAM). Notably both mevalonate cascade inhibition-induced autophagy and apoptosis were p53 dependent: simvastatin increased nuclear p53 accumulation, and both cyclic pifithrin-α and p53 shRNAi partially inhibited NOXA, PUMA expression and caspase-3/7 cleavage (apoptosis) and DRAM expression, Atg5-12 complex formation, LC3 lipidation, and autophagosome formation (autophagy). Furthermore, the autophagy response is induced rapidly, significantly delaying apoptosis, suggesting the existence of a temporally coordinated p53 regulation network. These findings are relevant for the development of statin-based therapeutic approaches in obstructive airway disease.

## Introduction

Apoptosis is an intrinsic cellular death response that occurs in the face of a myriad of extracellular insults. This complex process is the culmination of coordinately regulated intrinsic and extrinsic pathways involving the activation of intracellular pro-apoptotic effectors such as caspases, and modulation of pro- and anti-apoptotic Bcl-2 family members [1]. Autophagy is a dynamic process in which intracellular membrane structures sequester proteins and organelles for degradation in a lytic compartment. It is evolutionarily conserved, occuring in all eukaryotic cells [2], [3]. Autophagy reprocesses cellular components, contributing to organelle turnover and to the bioenergetic management of starvation [4]. During autophagy, parts of the cytoplasm (including whole organelles) are sequestered into double-membrane vesicles called autophagosomes. Autophagosomes ultimately fuse with lysosomes to generate single-membrane autophago-lysosomes that mediate the degradation of their contents [5]. A number of stimuli can induce autophagy, apoptosis or both; with concomitant induction, in a cell stimulus dependent manner autophagy can either protect against, or promote apoptosis, [6], [7], [8]. The molecular mechanisms that determine autophagy, apoptosis and their interaction are not fully established, but may involve induction of autophagy genes such as Atg5 in a cell type, stimulus, and cellular environment-specific manner.

In response to DNA damage, oncogenic activation, hypoxia or other forms of stress, p53 acts through transcription-dependent and -independent mechanisms to manage cellular responses that either stop or repair genomic damage to eliminate potentially oncogenic cells. The best-studied functions of p53 relate to its control of cell-cycle arrest and cell death [9], [10], [11]. A pro-apoptotic function of p53 occurs both at the level of transcription, through activation of proteins such as Puma, Noxa and Bax, and in the cytosol by binding anti-apoptotic proteins such as Bcl-2 and Bcl-XL [12], [13]. Autophagy induction by p53 may either contribute to cell death [6] or constitute a physiological cellular defense response [8]. As with apoptosis, the cellular localization of p53 modulates its impact in autophagy; cytosolic p53 inhibiting autophagy while nuclear p53 inducing and regulating autophagy through, for example, the transactivation of autophagy inducers such as DRAM, which encodes a lysosomal protein [6], [14], [15].

In the cholesterol synthetic pathway, the inhibition of 3-hydroxy-3-methyl-glutaryl-coenzyme A (HMG-CoA) reductase prevents the conversion of HMG-CoA to mevalonate, limiting the synthesis of cholesterol and its upstream intermediates such as the isoprenoids, farnesyl and geranygeranyl pyrophosphate (FPP and GGPP) [16]. Notably, FPP and GGPP are used as substrates for the prenylation of small GTP proteins, including Rho, Ras, Rac and Cdc42; a post-translational modification that is essential for the activation of these signaling effectors, thus enabling their critical roles in cell growth and survival [17], [18]. HMG-CoA reductase inhibitors such as statins can stimulate apoptosis in divergent somatic and cancer cells [19], [20]. Indeed, we recently showed that simvastatin induces apoptosis in human primary airway mesenchymal cells via a novel p53 dependent pathway involving selective release of Smac/Diablo and Omi/HtrA2 [13]. Notably, a recent study using hepatocytes indicates that some hydrophobic statins can also induce autophagy, but the precise mechanism for this effect was not elucidated clearly [21].

Here, we investigate the impact of mevalonate cascade inhibition on both autophagy and apoptosis, and dissect coordinating mechanisms for an interplay between these processes in primary human airway mesenchymal cells. Our studies indicate a role for p53 in promoting both autophagy and apoptosis, and reveal that autophagy is induced rapidly, providing an early protective response to statin-induced cell stress that leads to apoptosis.

## Materials and Methods

Cell culture plasticware was obtained from Corning Costar Co. (Canada). Cell culture media, propidium iodide (PI), simvastatin, mevalonate, cyclic Pifithrin-α, and 3-(4,5-dimethyl-2-thiazolyl)-2,5-diphenyl-2H-tetrazolium bromide) (MTT) were obtained from Sigma-Aldrich (Oakville, CA). Rabbit anti-human cleaved caspase-9, -7, -3, poly ADP-ribose polymerase (PARP), rabbit anti-human, PUMA, Bcl-2, Beclin-1, Atg-5, Atg-12 were purchased from Cell Signaling (Canada). Mouse anti-glyceraldehyde-3-phosphate dehydrogenase (GAPDH), rabbit anti-DRAM, and mouse anti-p53 were obtained from Santa Cruz Biotechnologies (USA). Rabbit anti-NOXA was obtained from Abcam (USA). Mitotracker Red, and Lysotracker Red were obtained from Invitrogen Molecular Probes (Canada). Casapase-Glo®-3/7 assay were purchased from Promega (USA). Rabbit-anti LC3-β, mouse-anti BNIP3 (Bcl-2/E1B-19K-interacting protein 3), Bafilomycin A1 (Baf-A1), 3-Methyl adenine (3-MA), were purchased from Sigma. Mouse anti-GAPDH, and rabbit anti-HDAC were purchased from Santa Cruz (US).

### Primary HASM and HAF cell culture preparation

For all experiments we used primary cultured human airway smooth muscle (HASM) cells and airway fibroblasts (HAF) that were prepared from 2^nd^ to 4^th^ generation bronchi in macroscopically healthy segments of resected lung specimens. After microdissection to separate the lamina reticularis and submucosal compartment from encircling airway smooth muscle bundle, both HAF and HASM cells were isolated by enzymatic dissociation as we have described [13], [22], [23]. All procedures were approved by the Human Research Ethics Board (University of Manitoba, ethic reference number:H2002:150). Cells were grown in Dulbecco's modified Eagle's medium supplemented with 10% fetal bovine serum with antibiotic. For all experiments cells were starved in 0.5% fetal bovine serum for 48 hrs prior to all treatments, which were also done in 0.5% fetal bovine serum. Medium was changed every 48 hrs. For all experiments, passages 3-7 of HASM and HAF were used.

### Cell viability assay

We measured cell viability of HASM and HAF under various treatment conditions, as we have described previously using 3-(4,5-dimethyl-2-thiazolyl)-2,5-diphenyl-2H-tetrazolium bromide (MTT) [24], [25]. Relative cell viability (percent of control) was calculated using the equation: (mean OD of treated cells/mean OD of control cells) ×100. For each time point, the treated cells were compared with control cells that had been treated with vehicle only (DMSO, 0.1% V/V).

### Measurement of apoptosis by flow cytometry

Apoptosis was measured using the Nicoletti method [7], [26]. Briefly, cells grown in 12-well plates were treated with 10 µM simvastatin for the indicated time intervals, alone or in presence of 3-MA, or Baf-A1. After scraping, the cells were harvested by centrifugation at 800 *g* for 5 min, washed once with phosphate-buffered saline, and resuspended in hypotonic propidium iodide lysis buffer (1% sodium citrate, 0.1% Triton X-100, 0.5 mg/ml RNase A, 40 µg/ml propidiume iodide). Cell nuclei were incubated for 30 min at 30°C and subsequently analyzed by flow cytometry. Nuclei to the left of the G1 peak containing hypo-diploid DNA were considered apoptotic.

### Luminescence caspase activity assays

Luminometric assays Caspase-Glo® -9 and -3/7 (Promega) were used to measure the proteolytic activity of caspase-3/7 (DEVD-ase), and -9 (LEHD-ase). Briefly, cells sub-cultured in 96-well plate at 15,000 cells/well, were treated with 10 µM simvastatin with or without inhibitors and harvested at different time points. Freshly prepared caspase reagents containing whole protein cell lysate extract buffer and either z-DEVD-luciferin, or z-LETD-Luciferin. In each experiment negative control cells (treated with vehicle only) were included. Plates were gently shaken at 300-500 rpm for 30 sec and incubated for 30 min at RT. The solution was then transferred to a white-well plate and the luminescence of each sample was measured using a luminometer.

### Sub-cellular fractionation

Cytosolic and nuclear fractions were generated using a digitonin-based subcellular fractionation technique at 4°C [7]. Cells were scraped, pelleted by centrifugation (800 g), then washed (PBS pH 7.2) and re-centrifuged. Pellets were permeabilized for 5 min on ice: 3×10^7^ cells/mL of cytosolic extraction buffer (250 mM sucrose, 70 mM KCl, 137 mM NaCl, 4.3 mM Na2HPO4, 1.4 mM KH_2_PO_4_ pH 7.2, 100 µM PMSF, 10 µg/ml leupeptin, 2 µg/ml aprotinin, containing 200 µg/ml digitonin). Plasma membrane permeabilization was confirmed by staining with 0.2% trypan blue solution. Cells were then pelleted (700 g, 20 min), and the supernatant was removed as the cytosolic fraction. Pellets were resuspended in the same volume of nuclear lysis buffer (50 mM Tris pH 7.5, 0.5 M NaCl, 2 mM EDTA, 0.1% SDS, 1% NP-40, 100 µM PMSF, 10 µg/ml leupeptin, 2 µg/ml aprotinin), followed by pelleting at 13,000 *g* for 3 min at 4°C, and this second supernatant collected as the nuclear fraction. For the detection of specific protein by immunoblotting, equal amounts of cytosolic and pellet fractions protein (calculated from the measured concentration of each extract) were supplemented with 5× SDS-PAGE loading buffer, subjected to standard 15% SDS-PAGE and transferred to nitrocellulose membranes.

### Stable gene silencing: lentiviral delivery of shRNA

The ATG5, and p53 shRNA constructs were obtained from Open Biosystems (ATG5: accession #NM_004849, clone V2LHS_195828; p53: accession #NM_000546 clone V2LHS_93613), as inserts in the lentiviral vector GIPZ distributed by the Manitoba Centre for Proteomics and Systems Biology as a plasmid in *E. coli* (DH5α). ATG7 lentiviral particles (sc-41447-V), and control shRNA lentiviral particles (sc-108080) were purchased from Santa Cruz Biotechnology (US). Individual colonies were amplified in LB broth with ampicillin (100 µg/mL), and purified using a QIAGEN Maxi-Prep Kit. A vesicular stomatitis virus G (VSVG) pseudo-typed lentiviral vector was made using HEK 293T cells by calcium phosphate transfection of purified ATG5 and p53 shRNA plasmid, virus packaging vector (8.2Δvpr), and a VSVG plasmid as described previously [13]. After 3 days the supernatant was collected and concentrated by ultra-centrifugation. For infection, primary HASM cells were grown to 70% confluence and transduced at a MOI of 6, in the presence of 8 µg/mL polybrene (final concentration), for 2 hours. Excess viral vectors were removed, and the transduced cells were cultured in fresh medium for 2 days before selection for stable expression of the shRNA by growing in culture media containing puromycin (4 µg/mL) for at least 3 weeks. For control cells, in tandem with preparation of ATG5 and p53 shRNAi lentivirus a GIPZ vector harboring “scrambled” non-coding shRNA was also prepared and used to generate lentivirus for transduction of the same primary HASM cell lines that were used to generate ATG5 and p53-deficient stable cultures. In separate experiments cells were directly infected with ATG7 shRNA and non-coding shRNA using lentivirus. Stable clones were selected using puromycin treatment. For ATG7 studies all procedures were performed according to manufacturer protocol (Santa Cruz Biotechnology, US).

### Immunoblotting

To prepare protein lysates cells were washed, and protein extracts prepared in lysis buffer (20 mM Tris-HCl (pH 7.5), 0.5% Nonidet P-40, 0.5 mM PMSF, 100 µM β-glycerol 3-phosphate and 0.5% protease inhibitor cocktail). After a high-speed spin (13,000 *g* ×10 min) supernatant protein content was determined by Lowry protein assay, then proteins were separated by SDS-PAGE and transferred to nylon membranes under reducing conditions. After blocking membranes with non-fat powdered milk and Tween 20, blots were incubated overnight with the primary antibodies at 4°C. Primary antibodies used included: cleaved caspase-3, -9, -7, PARP, PUMA, NOXA, p53, HDAC, LC3-β, beclin-1, BNIP3, Atg5-12, and GAPDH. HRP-conjugated secondary antibody incubation was for 1 hr at room temperature, and then blots were developed by enhanced chemiluminescence (ECL) detection (Amersham-Pharmacia Biotech).

### Immunocytochemistry, confocal imaging and electron microscopy

For immunocytochemistry, HASM and HAF cells were grown overnight on coverslips and then treated with simvastatin (10 µM) or vehicle for 72 hrs prior to fixation (4% paraformaldehyde/120 mM sucrose) and permeabilization (0.1% Triton X-100). Cells were incubated with rabbit anti-LC3-β (1∶250). The fluorescent images were then observed and analyzed using an Olympus FluoView multi-laser confocal microscope. Lysosomes were stained with 250 nM Lysotracker Red (Invitrogen Molecular Probes) before the cells were fixed [13].

For transmission electron microscopy (TEM), cells were fixed (2.5% glutaraldehyde in PBS, pH 7.4) for 1 h at 4°C, and then post-fixed in 1% osmium tetroxide before embedding in Epon. TEM was performed with a Philips CM10, at 80 kV, on ultra-thin sections (100 nm on 200 mesh grids) stained with uranyl acetate and counterstained with lead citrate.

### Quantitative RT-PCR for ATG5, PUMA, NOXA, and DRAM mRNA

Total cellular RNA was isolated using the RNeasy Plus Mini Kit (Qiagen, Mississauga, ON) then 1 µg was reverse transcribed using the QuantiTect Reverse Transcription Kit. The abundance of ATG5, PUMA, NOXA and DRAM mRNA were determined using the Applied Biosystems 7500 Real-Time PCR System thermocycler and the Power SYBR Green PCR Master Mix. Oligonucleotide primers were as follows: ATG5: forward, 5′- AGCCAATGTTGGAAACACCTCTGC-3′; reverse, 5′-TCCTTCAATCTGTTGGCTGTGGGA-3′, PUMA:forward:5′-CTGTGAATCCTGTGCTCTGC–3′; reverse: 5′- AATGAATGCCAGTGGTCACA – 3′,NOXA:forward: 5′-ATTACCGCTGGCCTACTGTG–3′, reverse: 5′- GTGCTGAGTTGGCACTGAAA –3′, DRAM:forward: 5′-ATTCCAGAGGAAGAAGCAGCCCTT–3′reverse: 5′-ACTTGGCCACACATGGGTTTATGC – 3′.

A dissociation curve was generated at the end of each PCR reaction to verify that a single product was amplified. 18S rRNA, primers 5′-CGCCGCTAGAGGTGAAATTC-3′ (forward) and 5′-TTGGCAAATGCTTTCGCTC-3′ (reverse) served as the endogenous reference gene. The relative expression levels of ATG5 normalised to 18S rRNA and relative to vehicle treated controls was calculated by the equation 2^(−ΔΔCt)^. The ΔC_T_ value was determined by subtracting the average 18s rRNA C_T_ value from the average C_T_ value of the corresponding target transcript. The calculation of ΔΔC_T_ values involves subtraction of the ΔC_T_ calibrator value (vehicle treated). For the vehicle treated samples ΔΔC_T_  = 0 and 2^0^ equals 1. For the simvastatin treated samples, 2^−ΔΔCT^ indicates the fold change in gene expression relative to time-matched controls.

### Statistical analysis

All results were expressed as mean ± SD and were compared by one-way or two-way ANOVA followed by Tukey's or Bonferroni's post hoc test, using Graph Pad Prism 4.0. *P*<0.05 was considered significant. Data were collected in triplicate from at least three cell lines unless otherwise indicated.

## Results

### Mevalonate cascade inhibition induces autophagy and apoptosis in human airway mesenchymal cells

**Figure 1:**
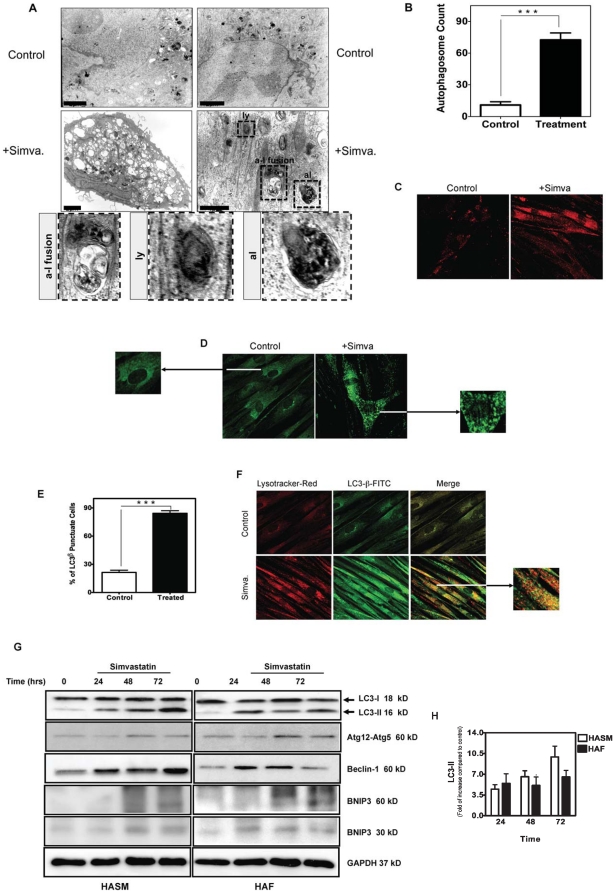
Simvastatin induces autophagy in primary human airway smooth muscle (HASM) cells and human airway fibroblasts (HAF). (A) HASM cells were either left untreated (*top panel*) or they were treated with 10 µM simvastatin (*lower panels*) for 72 hrs. Cells were then imaged by TEM. Magnification: 4.6×10^3^. Structures identified as autophagosomes are indicated with *black arrows.* Lysosomes (ly), fused autophagosomes and lysosomes (a-1 fusion) and late autophagolysosomes (al) are highlighted in magnified images of each cytosolic vesicle. The scale bar represents 2 micron in all the top row and left panel in the middle. The scale bar in the right side panel in the middle row represents to 1 micron. The lower row shows enlarged images of “ly”, “a-l fusion” and “al” regions highlighted by broken lines in the right hand panel of the middle row. (**B**) Quantification of classic autophagosomes (exclusive of large lucent vesicles seen in low magnification images of Fig. 1A) in six different views of TEM images in controls and simvastatin (10 µM, 72 hrs) treatments (with the same magnification) indicated significant difference of autophagosome between control and treatment groups (*P*<0.001*)* (**C**) HASM treated with simvastatin (+Simva, 10 µM, 72 hrs) showed increased in Lysotracker Red staining, a marker of lysosomal activation. (**D**) HASM treated with simvastatin (+Simva, 10 µM, 72 hrs) showed an increase in punctuate staining for LC3-β (*green*), a marker of autophagy. (**E**) Quantification of LC3β puncta in six different views of immunofluorescence images in controls and simvastatin (10 µM, 72 hrs) treatments (with the same magnification) indicated significant difference of LC3β puncta between control and treatment groups (*P*<0.001*)* (**F**) HASM cells treated with simvastatin showed an increase in co-localization of Lysotracker Red and LC3-β (*green*), indicating the fusion of lysosome and autophagosome to form an autophagolysosome. (**G**) Western blot analysis of cell lysates from HASM and HAF. Cells were treated with 10 µM simvastatin for the indicated time periods, and then immunoblotted using the indicated specific antibodies. BNIP3 appears as both a monomer (30 kDa) and a dimer (60 kDa). Glyceraldehyde 3-phosphate dehydrogenase (GAPDH) was used as loading control. (**H**) Densitometry analysis of LC3 II formation in HASM and HAF. Data represent means ± s.e. mean of three independent experiments, using 3 different cell lines. For each experiment LC3 II compared to control, and GAPDH was used as a loading control.

We have shown that simvastatin, an HMG-CoA reductase inhibitor, induces apoptosis in human airway mesenchymal cells by depleting mevalonate cascade intermediates [13]. Because autophagy and apoptosis may occur concurrently or sequentially in response to the same stimulus, we investigated whether simvastatin-induced cell death occurs in association with autophagy in human airway mesenchymal cells. Ultrastructural assessment using transmission electron microscopy of HASM cells treated with simvastatin (10 µM, 72 hrs), showed a significant increase in the formation of autophagosome-like vacuoles (Fig. 1A,B). Importantly, we also observed apoptosis in these experiments, consistent with the endpoints used in our previous studies [13] (Fig. S1A–C).

In autophagy lysosomes fuse with autophagosomes to form autophagolysosomes [7], [27], [28], thus, we next assessed changes in Lysotracker Red staining after simvastatin exposure (10 µM, 72 hrs). We observed a marked increase in lysosome number in HASM (Fig. 1C). LC3 exists in two forms: LC3-I and its lipidated derivative LC3-II; which localizes to autophagosomal membranes prior to their fusion with lysosomes [29]. In simvastatin-treated HASM cells we observed increased formation of LC3-punctae, a feature of autophagic cells (Fig. 1D, E). Furthermore, dual staining for LC3 and lysosomes revealed that simvastatin (10 µM, 72 hrs) induced their co-localization, an additional feature of autophagolysosome formation (Fig. 1F) and autophagic flux.

In both HASM and HAF we next investigated simvastatin-induced temporal changes in LC3 lipidation and abundance of other proteins involved in autophagosome formation. Ubiquitin-mediated association of Atg5 and Atg12 is required to recruit other proteins to the autophagosomal membrane and form the autophagic vacuole [7], [30], [31]. Concomitantly, beclin-1 is a part of an early complex that promotes biogenesis and growth of pre-autophagosomal membranes [32], [33]. Immunoblot data show that in human airway mesenchymal cells, simvastatin induced increased beclin-1, the appearance of LC3-II, and the formation of the Atg5-12 complex within 24–48 hrs (Fig. 1G). Quantitative densitometry of western blots for LC3 II revealed a rapid, >3.5 fold increase within 24 hours, a response maintained or that gradually increased thereafter in HAF and HASM, respectively (Fig. 1H). Notably, in the same samples, after 48 hrs we detected a marked increase in BNIP3 (Fig. 1I), an atypical pro-apoptotic Bcl-2 family member whose pro-apoptotic activity is distinct from that of other family members [34]. These data indicate that mevalonate cascade inhibition induced both autophagy and apoptosis, but the accumulation of proteins markers for each process was temporally disparate, with autophagy markers appearing in advance of those for apoptosis.

### Autophagy induced by simvastatin is blocked by mevalonate

**Figure 2:**
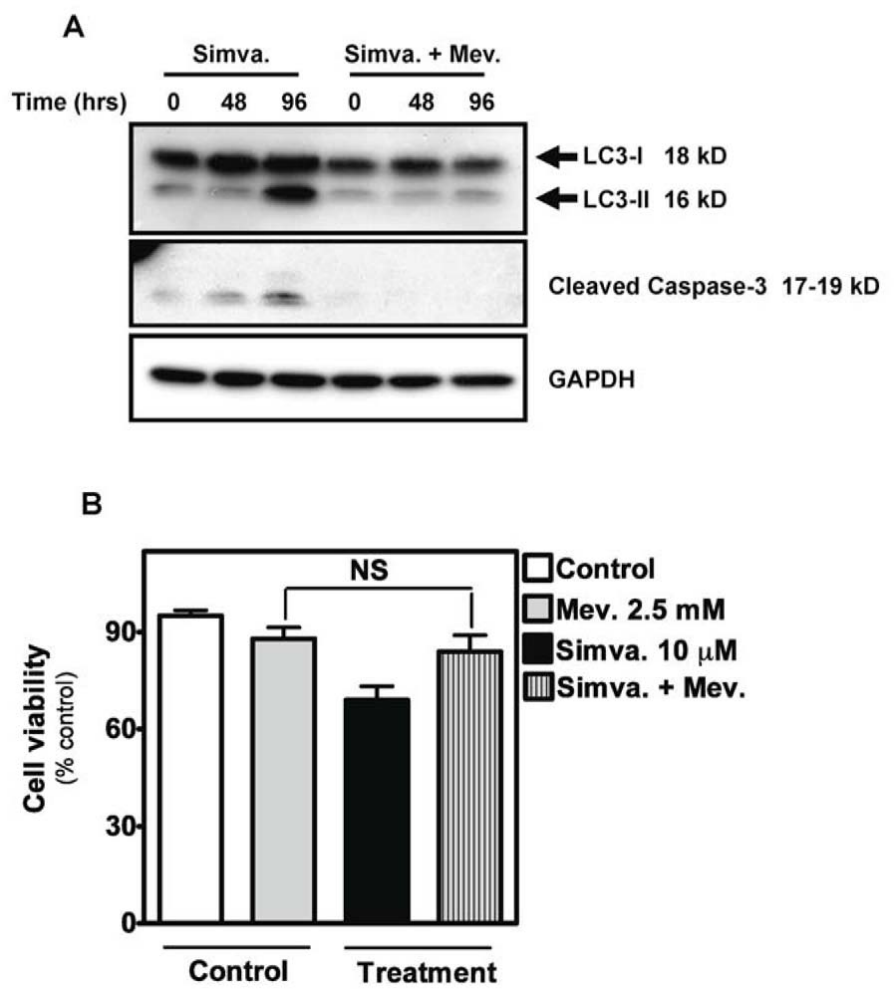
Mevalonate co-treatment inhibits simvastatin-induced apoptosis and autophagy. (**A**) Protein immunoblotting of cell lysates from HASM cells treated with 10 µM simvastatin with and without 2.5 mM mevalonate pretreatment, for the indicated time periods. Specific antibodies were used as indicated to detect levels of LC3-II, and the cleavage of caspases-3. GAPDH was used as loading control. (**B**) Cell viability of HASM cells measured following treatment with 10 µM simvastatin (Simva.) and/or 2.5 mM mevalonate (Mev.) as indicated. HASM were pretreated 4 hrs with indicated concentration of mevalonate and then co-treated for 96 hrs with simvastatin. Results are expressed as mean ± SD of 9 independent experiments using three different sources (donors) of HASM cells. NS, not significant.

We reported that addition of exogenous mevalonate is sufficient to reverse the pro-apoptotic effects of simvastatin in human airway mesenchymal cells [13]. Thus, we tested the effect of mevalonate addition on endpoints of both apoptosis and autophagy. Co-treatment with mevalonate (2.5 mM) prevented both LC3-II formation and caspase-3 activation (Fig. 2A), an effect that was associated with reduced simvastatin-induced cell death (Fig. 2B).

### Selective autophagy inhibition increases simvastatin-induced apoptosis

**Figure 3:**
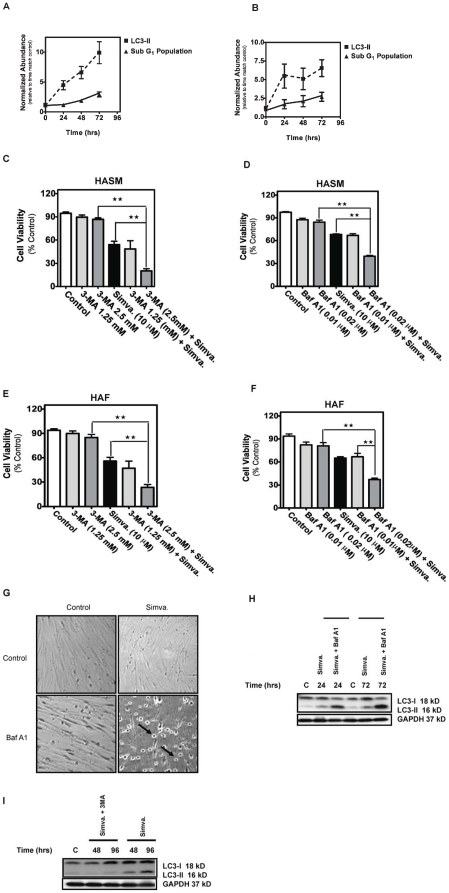
Autophagy inhibition increases simvastatin-induced cell death in HASM and HAF. (**A&B**) Side by side comparison of simvastatin-induced apoptosis (sub-G1 population of the cells) and autophagy (LC3 II formation) in HASM and HAF. Mevalonate cascade inhibition induced an early autophagy response and later apoptotic response. At each time point LC3-II level relative to its time matched control are normalized with time zero LC3-II levels to highlight temporal trends. Data represent means ± s.e. mean of three independent experiments, using 3 different cell lines. Cell viability was measured in cultured HASM and HAF after treatment with 10 µM simvastatin for 96 hrs with and without pretreatment with the following inhibitors of autophagy: (**C, E**) 1.25 or 2.5 mM 3-MA; (**D, F**) 0.01 or 0.02 µM Baf-A1. Results are expressed as percentage of corresponding time point control and represent the means ± SD of 12 independent experiments in three different sets of donor-matched HASM and HAF **, *P*<0.01; ***, *P*<0.001. (**G**) HASM cells co-treated with 10 µM simvastatin for 48 hrs with and without 0.02 µM Baf-A1, photographed under phase contrast microscopy settings. Arrows indicates partially detached cells with condensed morphology.(**H**) Baf-A1 enhanced LC3-II level in simvastatin treated cells. HASM were treated with simvastatin (10 µM) in indicated time points in presence and absence of Baf-A1. Baf-A1-simvastatin increased LC3-II level compared to simvastatin treatment. (**I**) 3-MA decreased LC3-II level in simvastatin treated cells. HASM were treated with simvastatin (10 µM) in indicated time points in presence and absence of 3-MA. 3-MA-simvastatin increased LC3-II level compared to simvastatin treatment.

Mevalonate cascade inhibition rapidly induced LC3βII formation, a marker of autophagy, while apoptosis induction was much less marked during the same 72 hr treatment period for both HASM and HAF (Fig. 3A&B).

Autophagy induced by some cytotoxic agents is lethal to target cells, but can also act as a survival mechanism that provides constituents necessary for supporting cell metabolism in various stress conditions [7], [24], [35], [36], [37], [38], [39], [40]. To investigate the relationship between mevalonate cascade inhibition-induced cell death and autophagy, we used a number of interventions to inhibit autophagy then assessed effects on cell viability. Cells were co-treated with simvastatin and one of two chemical inhibitors: (*i*) 3-methyladenine (3-MA), a class III PI3 kinase inhibitor, and (*ii*) bafilomycin-A1 (Baf-A1), a specific lysosomal vacuolar-type H^+^-ATPase pump inhibitor that blocks the fusion of autophagosomes and lysosomes [7], [27]. Dose response experiments were done for both inhibitors to find concentrations that inhibited autophagy without affecting viability in control HASM and HAF cells. In HASM and HAF each inhibitor suppressed simvastatin-induced autophagy and resulted in more rapid and significantly greater degree of cell death (*P* <0.01, Fig. 3C–F). Concomitant with reduced viability we observed morphological features of apoptosis (e.g., cell rounding, shrinkage, partial detachment) in HASM co-treated with simvastatin and autophagy inhibitors (Fig. 3G).

To characterize the effects of simvastatin on autophagic flux we assessed the impact of co-treatment with inhibitors of early (3-MA) and late (Baf-A1) autophagy on LC3-II formation. An increase in LC3-II protein, LC3 puncta and autophagosomes could result from blockade of autophagy at a late stage (lysosome-autophagosome fusion and lysosome function) [7], [29]; thus we measured LC3-II in the presence of both Baf-A1 and simvastatin in HASM (Fig. 3H) and found LC3-II levels augmented, indicating that simvastatin-induced-LC3-II formation was not the result of the blockage of autophagolysosme formation. As 3-MA inhibits early autophagy events [7]we also assessed its impact when added with simvastatin on autophagy flux, and found it decreased simvastatin induced LC3-II levels in HASM (Fig. 3I).

**Figure 4:**
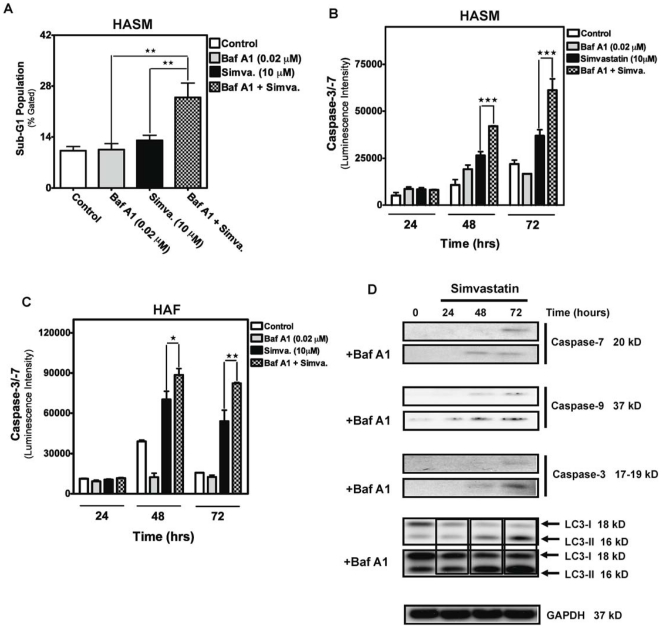
Autophagy inhibition increases apoptosis in HASM and HAF treated with simvastatin. (**A, B**). HASM were pretreated with 0.02 µM Baf-A1 in combination with 10 µM simvastatin for 96 hrs and the sub-G1 population was quantified using the Nicoletti method. Results shown are mean ± SD of 3 independent experiments in HASM primary cell lines from two different donors. **, *P*<0.01, compared to time-matched control. (**B&C**) Measurement of caspase activity in HASM and HAF co-treated with 10 µM simvastatin and 0.02 µM Baf-A1. Caspase-3/-7 activity were measured in treated cells at the indicated time points and compared to time-matched controls. ***, *P*<0.001. (**D**) Protein immunoblots used to access levels of cleaved caspases as well as the appearance of LC3-II in HASM treated with 10 µM simvastatin and 0.02 µM Baf-A1, for the indicated time periods. Detection of GAPDH served as a loading control.

To further characterize the relationship between autophagy and apoptosis in our experimental system we focused on other endpoints of cell death. Baf-A1 inhibition of simvastatin-induced autophagy resulted in increased numbers of HASM in the sub-G1 DNA compartment (*P*<0.01, Fig. 4A). Furthermore, we found that pretreatment with Baf A1 significantly increased simvastatin-induced caspase-3/-7 activity (Fig. 4B-D). Using protein immunoblotting we also found that autophagy inhibition, using Baf-A1, resulted in earlier and increased simvastatin-induced cleavage of caspases-3, -7 and -9 (Fig. 4B-D). Importantly, Baf-A1, which targets autophagosomes-lysosome fusion, promoted the accumulation of LC3-II (Fig. 4D), confirming a functional requirement for lysosomes in simvastatin-induced autophagy.

**Figure 5:**
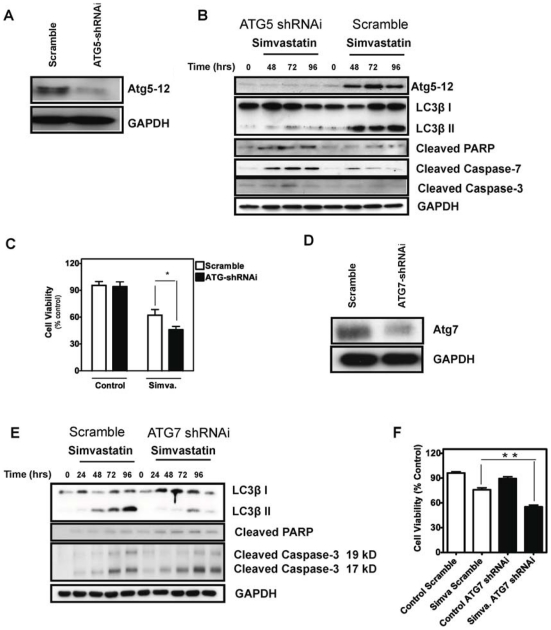
Inhibition of Atg5 and Atg7 by specific shRNAi resulted in an increase of cell death in simvastatin-treated HASM. (**A**) Protein immunoblotting demonstrate that ATG5 shRNAi significantly inhibited Atg5-12 complex formation. Scrambled sequence was used as an RNAi control. (**B**) Protein immunoblotting of simvastin-treated HASM, both control and ATG5 shRNAi. Specific antibodies against the indicated proteins were used, with GAPDH serving a loading control. (**C**) Cell viability assay (MTT assay) using control and ATG5 shRNAi HASM cells, with and without simvastatin treatment (10 µM, 96 hrs) showed that ATG5 shRNAi significantly increased simvastatin induced cell death (*P<*0.05). (**D**) Protein immunoblot demonstrating that ATG7 shRNAi significantly inhibited Atg7 expression. Scrambled sequence was used as an RNAi control. (**E**) Immunoblotting of simvastatin-treated HASM after infection with lentivirus harboring control or ATG7 shRNAi. Specific antibodies against the indicated proteins were used, with GAPDH serving a loading control. (**F**) Cell viability assay (MTT assay) using control and ATG7 shRNAi HASM cells, with and without simvastatin treatment (10 µM, 96 hrs) showed that ATG7 shRNAi significantly increased simvastatin induced cell death (*P<*0.01).

To rule out non-selective effects of chemical inhibitors, we also used shRNAi in HASM cells to silence Atg5 expression, which resulted in a significant inhibition of Atg5-12 formation Fig. 5(A). The silencing of Atg5 also accelerated apoptosis and decreased autophagy flux, as indicated by both an increase in the cleaved forms of caspases-3, -7 and PARP and decreased LC3-II levels compared to cells expressing control scrambled shRNAi (Fig. 5B). Importantly, in Atg5-silenced HASM cells simvastatin toxicity was significantly increased (*P*<0.05, Fig. 5C). ATG7 shRNA (Fig. 5D) was also used to more rigorously assess the impact of chemical inhibitors and ATG5 silencing. In a manner similar to these interventions, we found that ATG7 knock down increased simvastatin-induced caspase-3 activity, PARP cleavage and cell death (Fig. 5E&F). Collectively, our data reveal that autophagy induced by mevalonate cascade inhibition provides an early negative modulator signal for subsequent cellular apoptosis.

### p53 is a regulator of simvastatin-induced apoptosis and autophagy

**Figure 6:**
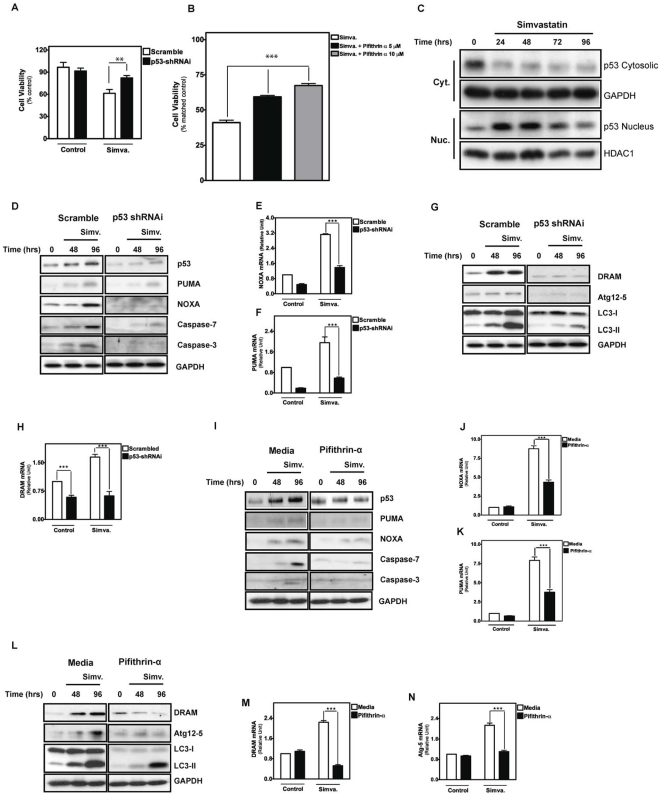
Simvastatin-induced apoptosis and autophagy are mediated via p53-dependent pathway. (**A**) Cell viability assay carried out on cells transduced with shRNAi targetting p53 or scrambled sequence as a control, with and without treatment with 10 µM simvastatin for 96 hours. Results are expressed as percentage of corresponding control and represent mean ± SD of 6 independent experiments (**: *P*<0.01). (**B**) HASM assesses using MTT assay. Cell viability was measured by comparing each treatment with corresponding control. Results reflect mean ± SD of four independent experiments (* * *: *P*<0.001). (**C**) Immunoblotting of cytosolic (Cyt) and nuclear (Nuc) extracts from HASM cells treated with 10 µM simvastatin for the indicated time points, using p53 antibody to detect nuclear translocation. HASM were treated with simvastatin (10 µM) for indicated time points (0–96 hrs). Glyceraldehyde 3-phosphate dehydrogenase (GAPDH) and histone deacetylase 1 (HDAC1) were used as loading controls for cytoplasmic and nuclear fraction, respectively. (**D**) Immunoblots of effects of p53 shRNAi silencing on apoptosis markers. HASM cells stably infected with scrambled shRNAi (left panels) and p53 shRNAi (right panels) were treated with simvastatin (10 µM) for indicated time points (0–96 hrs). NOXA, PUMA, and cleaved caspase-3 and -7 abundance were measured. (**E, F**) Quantitative RT-PCR to demonstrate the effect of p53-shRNAi on PUMA (E) and NOXA (F) expression. Results reflect mean ± SD of three independent experiments (* * *: *P*<0.001). (**G**) Immunoblots of effects of p53 shRNAi silencing on autophagy markers. HASM cells stably infected with scrambled shRNAi (left panels) and p53 shRNAi (right panels) were treated with simvastatin (10 µM) for indicated time points (0–96 hrs). DRAM, ATG-Atg12, and LC3-I and –II were assessed. (**H**) Quantitative RT-PCR to demonstrate the effect of p53-shRNAi on DRAM expression. Results reflect mean ± SD of three independent experiments (* * *: *P*<0.001). (**I**) Immunoblotting of cell lysates from cells treated with 10 µM simvastatin for the indicated time periods in the presence or absence of the p53 inhibitor pifithrin-α, using antibodies specific for the indicated proteins. GAPDH was used as loading control. (**J, K**) Quantitative RT-PCR to demonstrate the effect of pifithrin-α on NOXA (J) and PUMA (K) expression. Results reflect mean ± SD of three independent experiments (* * *: *P*<0.001). (**L**) Immunoblotting of cell lysates from cells treated with 10 µM simvastatin for the indicated time periods in the presence or absence of the p53 inhibitor pifithrin-α, using antibodies specific for the indicated proteins. GAPDH was used as loading control. (**M, N**) Quantitative RT-PCR to demonstrate the effect of pifithrin-α on DRAM (N) and Atg5 (N) expression. Results reflect mean ± SD of three independent experiments (* * *: *P*<0.001).

We previously showed that apoptosis induced by mevalonate cascade inhibition in human airway mesenchymal cells is p53-dependent [13]. Since p53 can regulate autophagy and apoptosis [15], we next suppressed p53 activity in HASM cells using shRNAi or the chemical inhibitor, cyclic pifithrin-α, and measured the impact on cell death. Regardless of the method of intervention, suppression of p53 led to significantly increased cell viability after treatment with simvastatin (*P*<0.01, Fig. 6A, *P*<0.001, Fig. 6B). We monitored the sub-cellular localization of p53 and found that mevalonate cascade inhibition promoted its redistribution to the nucleus after 24 hours, an effect sustained for several days (Fig. 6C). We also assessed expression of markers of simvastatin-induced apoptosis and found that silencing of p53 or treatment with pifithrin-α inhibited PUMA and NOXA induction with parallel inhibition of caspase-3 and -7 cleavage (Fig. 6D–F and I–K). In concert, we also assessed the impact of p53 inhibition on autophagy markers and observed that DRAM expression, Atg5-12 complex formation, and LC3-β II formation was reduced (Figs. 6G,H, L–N). These data show that both apoptosis and autophagy induced by mevalonate cascade inhibition are regulated by p53. Thus, p53 plays a dual role, being required for both simvastatin-induced autophagy and apoptosis.

## Discussion

We have reported that inhibition of the mevalonate cascade in primary human HASM and HAF induces apoptosis via a novel p53-dependent pathway which involves inhibition of small Rho-GTPase anchorage to the cytoplasmic membrane without changing total cellular cholesterol content [13]. We now extend these findings and show that concurrent with apoptosis, mevalonate cascade suppression by HMG-CoA reductase inhibition induces an early p53-dependent autophagic response that appears to be a mechanism for quenching rapid apoptotic cell death that results from simvastatin-induced cell stress. An increasing body of evidence supports the existence of cross-talk between apoptosis and autophagy, including both positive and negative interactions [7], [8], [13], [41], [42], [43]. To our knowledge, this is the first report highlighting the interplay between autophagy and apoptosis during conditions that inhibit protein lipidation cascades that regulate intracellular signaling.

Autophagy has been viewed both as a cell-survival, and as a cell death response, but discriminating both roles is difficult in some experimental systems [44]. The existence of autophagic vesicles in dying cells may suggest a causal relationship between autophagy and cell death [45], [46], or it may represent remnants from an initial adaptive reaction to support cell survival under stress [47], [48]. In the present study, co-treatment of HASM and HAF with the autophagy inhibitors 3-MA, and Baf-A1 markedly increased simvastatin-induced activation of caspase-3 and -7 and cell death, suggesting a protective (pro-survival) role for autophagy. A protective role for autophagy is further supported by our observation that simvastatin-induced cell death was significantly increased in HASM cells in which the Atg5 expression had been silenced. Furthermore, Atg5 silencing was associated with increased activity of caspase-3, -7 and also PARP cleavage upon simvastatin treatment. The protective role for autophagy against simvastatin-induced cell death suggests that autophagy is an important physiological pro-survival process under stress conditions induced by inhibiting synthesis of isoprenoid lipid anchors required for activation of small GTPases.

LC3 lipidation is currently one of the most reliable markers of autophagosome formation in mammalian cells, where the relative amount of LC3-II reflects autophagosome abundance [7], [29]. Autophagic cells are also characterized biochemically by the presence of cleaved LC3, and its punctuate redistribution inside the cell [7]. We observed punctuate pattern of LC3-distribution in human airway mesenchymal cells, upon treatment with simvastatin, and it colocalized to lysosomes. In mammalian cells, autophagosomes form in the cytosol and then they fuse with lysosomes for degradation [29]. Both processes are influenced by distinct control mechanisms [7], [29]. Cellular LC3-II correlates with autophagosome number in mammalian cells [29], whereas an increase in the level of LC3-βII, as observed in our study upon simvastatin treatment, is mostly a result of enhanced autophagosomal formation, inhibited autophagosomal degradation, or a combination of the two [29]. In the present study, we observed that pretreatment with 3-MA was associated with a decrease in simvastatin-induced LC3-βII formation in airway mesenchymal cells, essentially reversing the effect of simvastatin and blocking autophagy. In contrast, pretreatment with the vacuole H^+^-ATPase inhibitor Baf-A1, which blocks later autophagosomal degradation, *increased* the formation of LC3-βII in simvastatin-treated airway mesenchymal cells up to 72 hrs after treatment. These studies suggest that simvastatin induces autophagy by enhancing the synthesis of autophagosomes. Notably, the use of Baf-A1 resulted in a corresponding increase in caspase-3, -7, and -9 cleavage and activation, indicating increased apoptotic signaling. We thus conclude that simvastatin-induced autophagy in human airway mesenchymal cells involves enhanced autophagosomal synthesis and may be a modulator mechanism for apoptosis.

We and others have shown that simvastatin-induced apoptosis can be reversed by the downstream product, mevalonate, demonstrating that simvastatin's action is mediated by changes in protein prenylation [13], [49], [50]. In the present study, we showed that simvastatin-induced autophagy was inhibited by mevalonate co-treatment, confirming that mevalonate cascade inhibition could induce both apoptosis and autophagy. We have previously shown that apoptosis triggered by mevalonate cascade inhibition is p53-dependent [13]. In those experiments, simvastatin increased the expression of PUMA and NOXA, as well as the translocation of these proteins to mitochondria [13]. Others observed a similar involvement of p53 in the inhibition of cell proliferation in different cell types [51], [52]. The present study extends our previous work and demonstrates a role for p53 in simvastatin-induced autophagy, and the interplay between autophagy and apoptosis upon simvastatin treatment. The use of either pifithrin-α or p53 shRNAi significantly inhibited simvastatin-induced cell death in HASM. There are several reports indicating that p53 regulates autophagy in other biologic systems [6], [8], [12], [14], [53]. For example, p53 activation-induced autophagy may be mediated by transactivation of autophagy inducers such as DRAM [6]. In this study we show by immunoblotting and qRT-PCR that simvastatin treatment induced DRAM expression in HASM cells, along with the p53-induced apoptotic proteins PUMA and NOXA. Inhibition of p53 with either pifithrin-α or p53-shRNAi resulted in the loss of simvastatin-induced PUMA, NOXA and DRAM expression followed by reduced caspase cleavage (marker of apoptosis) and LC3-II formation (marker of autophagy) compared to wild type or scramble shRNAi infected cells treated with simvastatin. Others have reported a cellular localization-dependence effect of p53: cytosolic p53 would inhibit autophagy while nuclear p53 is pro-autophagic [12]. Our results showed that simvastatin treatment provoked p53 translocation to nucleus. Thus, our data is in agreement with recent findings that nuclear p53 can induce both autophagy and apoptosis through transcriptional effects [12].

It has been previously reported that depolarized mitochondria are rapidly eliminated by autophagy in primary hepatocytes, leading to the hypothesis that autophagy may protect against apoptosis by increasing the threshold of mitochondria-dependent cell death [54]. In this model, elimination of damaged mitochondria by autophagy might prevent the release of proapoptotic signals from mitochondria [55]. In the absence of this scavenging process, the release of reactive oxygen species from damaged mitochondria would contribute to apoptotic-, and in severe cases, necrotic cell death. In our experimental system, inhibition of simvastatin-induced autophagy enhanced simultaneously-occurring apoptotic cell death pathways. It is unclear whether autophagic elimination of depolarized mitochondria may be stimulated by mevalonate cascade inhibition, but a number of reports by us and others have revealed that mitochondrial ROS production can be induced by statin exposure in vitro [13], [56], [57].

The data presented here show that mevalonate cascade inhibition in human airway mensenchymal cells leads to activation of both apoptotic and autophagic responses, with the latter effect occurring more rapidly and counteracting the former for a finite duration; in other words, p53-induced autophagy appears to be a negative regulator of p53-induced apoptosis. Our data also indicate that both direct mechanisms (p53-dependent upregulation of DRAM), as well as indirect effects (i.e. BNIP3-mediated reversal of inhibitory effect of Bcl2 on beclin-1) may form the molecular foundation of p53-proautophagic effect triggered by simvastatin.

## Supporting Information

Figure S1 **Simvastatin induces apoptosis in primary human away smooth muscle (HASM) cells and airway fibroblasts (HAF).** (**A**) The cells were treated with simvastatin (10 μM) and cell viability was assessed 48 and 96 hrs thereafter by MTT assay. Control cells for each time point were treated with the solvent control (DMSO). Results are expressed as percentage of corresponding time point control and represent the means ± SD of 12 independent experiments in three different sets of patient-matched HASM and HAF (**, *P*<0.01; ***, *P*<0.001). (**B**) HASM and HAF cells were treated with simvastatin (10 µM) and at the indicated time points apoptosis was measured using Nicolleti method (see materials and methods). Percent sub-G1 HASM and HAF abundance induced by simvastatin or DMSO solvent control after 48 and 96 hrs. Results represent the means ± SD of 6 independent experiments in two different patient-matched HASM and HAF primary cell lines. **, *P*<0.01; and ***, *P*<0.001 compared to time-matched control. (**C**) Effects of simvastatin (10 μM) treatment (24 and 72 hrs) on caspase-3/-7, and caspase-9 enzymatic activity, as detected by Caspase-Glo® luminometric assay. Caspase activity normalized to that measured for solvent-only treated cultures is represented on the Y-axis. The data represent mean ± SD of duplicate experiments performed on 4 different patient-matched primary HASM and HAF cell lines.
(TIFF)
