# Supplementary figures and images for: Mevalonate Cascade Regulation of Airway Mesenchymal Cell Autophagy and Apoptosis: A Dual Role for p53
Source: PLoS One. 2011 Jan 31;6(1):e16523. doi: 10.1371/journal.pone.0016523 (PMC3031577; doi:10.1371/journal.pone.0016523)

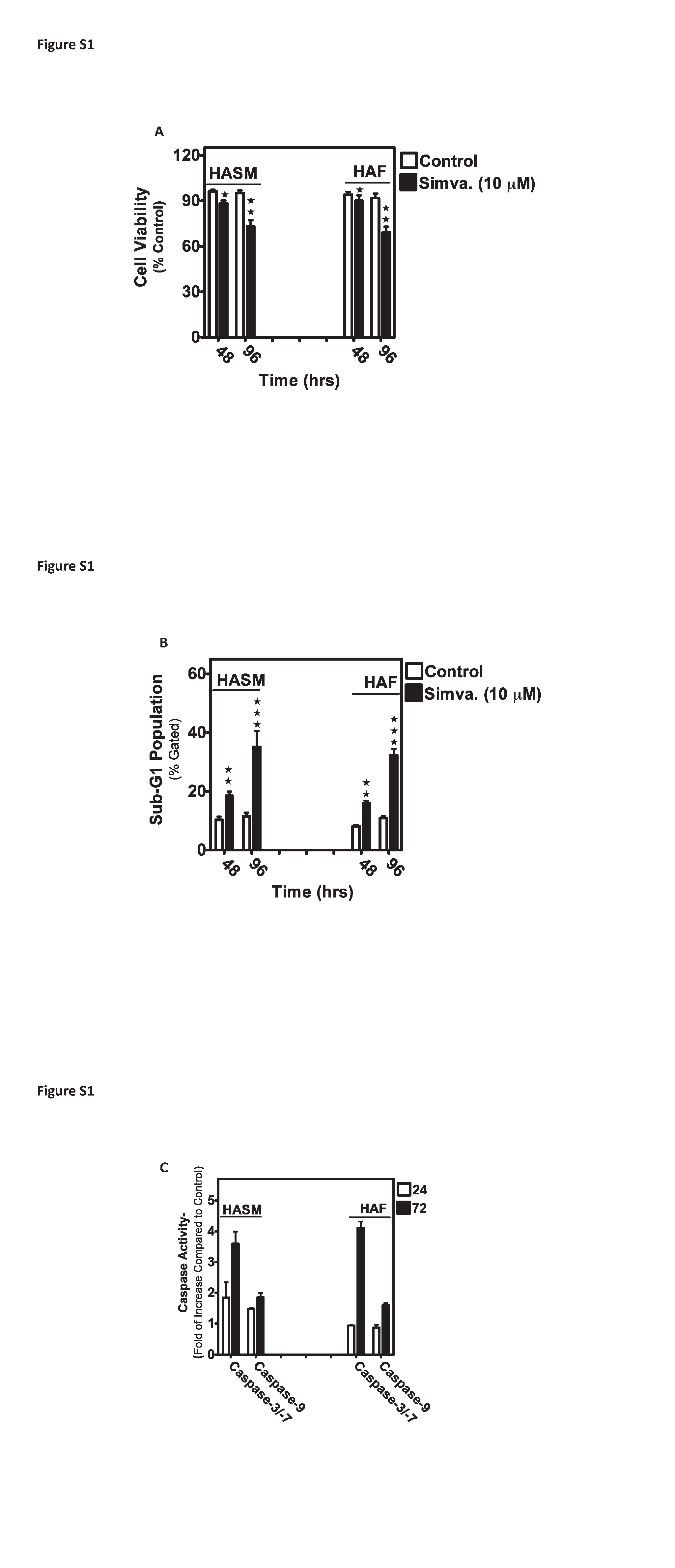

Supplement: Figure S1 — Simvastatin induces apoptosis in primary human away smooth muscle (HASM) cells and airway fibroblasts (HAF). (A) The cells were treated with simvastatin (10 μM) and cell viability was assessed 48 and 96 hrs thereafter by MTT assay. Control cells for each time point were treated with the solvent control (DMSO). Results are expressed as percentage of corresponding time point control and represent the means ± SD of 12 independent experiments in three different sets of patient-matched HASM and HAF (**, P<0.01; ***, P<0.001). (B) HASM and HAF cells were treated with simvastatin (10 µM) and at the indicated time points apoptosis was measured using Nicolleti method (see materials and methods). Percent sub-G1 HASM and HAF abundance induced by simvastatin or DMSO solvent control after 48 and 96 hrs. Results represent the means ± SD of 6 independent experiments in two different patient-matched HASM and HAF primary cell lines. **, P<0.01; and ***, P<0.001 compared to time-matched control. (C) Effects of simvastatin (10 μM) treatment (24 and 72 hrs) on caspase-3/-7, and caspase-9 enzymatic activity, as detected by Caspase-Glo® luminometric assay. Caspase activity normalized to that measured for solvent-only treated cultures is represented on the Y-axis. The data represent mean ± SD of duplicate experiments performed on 4 different patient-matched primary HASM and HAF cell lines. (TIFF) [file pone.0016523.s001.tif]
